# Bifocal and Multifocal Contact Lenses for Presbyopia and Myopia Control

**DOI:** 10.1155/2020/8067657

**Published:** 2020-03-27

**Authors:** Laura Remón, Pablo Pérez-Merino, Rute J. Macedo-de-Araújo, Ana I. Amorim-de-Sousa, José M. González-Méijome

**Affiliations:** ^1^Department of Applied Physics, University of Zaragoza, 50009 Zaragoza, Spain; ^2^Department of Ophthalmology, Biomedical Research Institute Fundación Jiménez Díaz, 28040 Madrid, Spain; ^3^Clinical and Experimental Optometry Research Lab (CEORLab), Center of Physics, University of Minho, 4710-057 Braga, Portugal

## Abstract

Bifocal and multifocal optical devices are intended to get images into focus from objects placed at different distances from the observer. Spectacles, contact lenses, and intraocular lenses can meet the requirements to provide such a solution. Contact lenses provide unique characteristics as a platform for implementing bifocality and multifocality. Compared to spectacles, they are closer to the eye, providing a wider field of view, less distortion, and their use is more consistent as they are not so easily removed along the day. In addition, contact lenses are also minimally invasive, can be easily exchangeable, and, therefore, suitable for conditions in which surgical procedures are not indicated. Contact lenses can remain centered with the eye despite eye movements, providing the possibility for simultaneous imaging from different object distances. The main current indications for bifocal and multifocal contact lenses include presbyopia correction in adult population and myopia control in children. Considering the large numbers of potential candidates for optical correction of presbyopia and the demographic trends in myopia, the potential impact of contact lenses for presbyopia and myopia applications is undoubtedly tremendous. However, the ocular characteristics and expectations vary significantly between young and older candidates and impose different challenges in fitting bifocal and multifocal contact lenses for the correction of presbyopia and myopia control. This review presents the recent developments in material platforms, optical designs, simulated visual performance, and the clinical performance assessment of bifocal and multifocal contact lenses for presbyopia correction and/or myopia progression control.

## 1. Introduction

Bifocal and multifocal contact lenses (CLs) for presbyopia correction and/or myopia control can be made available in a wide variety of platforms, including rigid gas permeable (RGP) lenses of different sizes from corneal to scleral supported, soft contact lenses, and hybrid lenses [1]. In addition to the lens optical structure, bifocality and multifocality can also be achieved by reshaping the cornea with the application of CLs in the technique called orthokeratology and has been used widely for myopia correction and myopia control [2, 3] although its application for presbyopia correction is still limited [4]. While presbyopia correction with contact lenses accounts for up to 25–35% of the contact lens fittings in several countries [5], myopia control contact lens fittings are still limited to 2–5% of the contact lenses fitted [6].

The pupil size of the eye and the power distribution across the lens are related to providing the desired effect for presbyopia and myopia applications [1, 2, 7]. In presbyopia correction, the main goal is to provide images focused at different distances along the optical axis and is, therefore, a matter of central (foveal) viewing. For older patients senile miosis imposes a limitation of the area of the device that is useful to form images in the retinal proximity. However, in the case of myopia control, in addition to foveal imaging, off-axis imaging should also be taken into account as it could be relevant to achieve the therapeutic effect and slow eye growth (yet to be confirmed) [8].

In this review article, we present an overview of the recent developments of bifocal and multifocal contact lens designs for the correction of presbyopia and myopia control, including the optical design of different platforms for bifocality and multifocality, computational simulations and performance assessment, and their connection with the visual performance, patient acceptance, and efficacy. For further information on the performance of earlier designs for presbyopia correction [1, 9] and myopia control, including orthokeratology [10, 11], the reader must consult the abundant existing literature including several systematic reviews and meta-analyses on the former topic [12–16].

## 2. Platforms for Bifocality and Multifocality in Contact Lenses

Contact lenses that allow the lens to change the relative position with the pupil depending on the viewing distance are mostly built in RGP platforms. Although segmented bifocal spectacles have been used for myopia control, alternating bifocal contact lenses have not been used for such purpose. Contact lenses whose optical zone remains stable regarding the pupil on different eyesight directions can be built in any platform from corneal to scleral RGP, soft and hybrid materials.

Most of the more effective bifocal and multifocal contact lens designs are currently manufactured in soft platforms. Due to its larger diameter and flexibility, it enables better control of centration and lens movement compared to corneal RGP contact lenses. Recently, these multifocal designs that have been introduced on hybrid (in which the central area of the contact lens is manufactured with rigid gas permeable materials) and scleral lenses also offer an excellent solution for presbyopia compensation and myopia progression control.

While soft, corneal RPG, and hybrid contact lens platforms have been the object of previous reviews [1], multifocal scleral supported contact lenses have been recently used for presbyopia correction. Modern RGP scleral lenses have a large diameter, without any mechanical interactions between the lens, the cornea, and the sclerocorneal limbus. Scleral contact lenses (SL) are considered as one of the best visual correction options for eyes that were unsuccessful with conventional contact lens modalities, which led to an exponential increase in the number of publications in the last years [17]. Progress in the manufacturing process, lens materials, and improved knowledge on the scleral anatomy boosted the indications for SL fitting. SL are mostly fitted to improve vision in cases of irregular astigmatism (from primary corneal ectasias to keratoplasty) and for providing a therapeutic environment for managing severe anterior eye diseases (severe dry eye due to Sjögren's or Stevens–Johnson syndrome) and also for normal/healthy corneas with high refractive errors [18–21].

The optical principles for scleral lenses are identical to corneal RGP and hybrid contact lenses, as corneal astigmatism (regular or irregular) and high-order aberrations are partially or completely compensated by the tear film reservoir between the lens and the cornea. However, SL wearers and manufacturers could also take advantage of the unique stability on-eye during lens wear: these lenses are rotationally stable and have lack of movement with blinking [22, 23].

Although SL are very stable on-eye, they tend to decenter. The geometric characteristics of the ocular surface beyond the corneal borders (flatter sclera in the nasal side), gravity, and eyelids effect usually make the SL to decenter inferotemporally [24–29]. However, some manufacturers are able to overcome this issue by decentering the optic zone to compensate for this misalignment with the visual axis, which could be very beneficial for presbyopic and myopia control designs. Nowadays, multifocal SL account for approximately 2% of all contact lenses prescribed [30]. Several SL designs have been introduced to the market in the last few years, with parameters varying considerably between manufacturers (center distance or near designs, different central optic zone diameter, addition powers, and power profiles), which enhance the importance to follow the fitting guides and recommendations [31].

## 3. Optical Designs

### 3.1. Bifocal and Multifocal Contact Lenses

**Figure 1:**
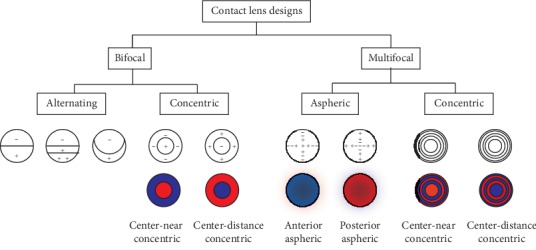
Illustration of different contact lens designs. In red: areas for near vision; in blue: areas for distance vision.

There are different bifocal and multifocal contact lens designs commercially available [32–34]. The International Organization for Standardization (ISO) [35] Ophthalmic Optics-Contact Lenses, Part 1: Vocabulary, Classification System and Recommendations for Labelling Specifications. ISO 18369-1:2006 (Geneva, Switzerland: ISO; 2006) defined the following concepts related to the matter of this review article as (i) *bifocal contact lenses*: contact lens designed with two optic zones, usually for distance- and near-vision correction, (ii) *multifocal contact lens*: contact lens designed to provide two or more zones of different refractive power, and (iii) *progressive power contact lens/varifocal power contact lens*: contact lens designed to provide correction for more than one viewing range in which the refractive power changes continuously, rather than discretely. Most of these contact lens designs can also be designed with toric geometry for the correction of astigmatism, particularly for rigid gas permeable lenses and also for some hydrophilic soft contact lenses.  Figure 1 shows different examples of multifocal contact lens designs.

These design concepts work under two different principles [9, 32, 36]: (i) *alternating image*, in which a translating movement of the lens when looking downwards results in viewing through an area with a different refractive power; and (ii) *simultaneous image,* where the simultaneous projection of the images coming from multiple target distances are presented to the eye at the same time at different focal planes. Then, in the simultaneous image, there must be a neural adaptation to select the sharp image depending on the visual target.

### 3.2. Simultaneous Image Contact Lens Designs

In simultaneous image designs, specific regions of the contact lens are designed for far and near vision correction, refracting simultaneously light from far and near targets through the pupil for all gaze positions. In this situation, the retina receives several images: in-focus and out-of-focus. Thus, lens centration, pupil size, ocular optics, and neural adaptation are essential for efficient visual performance with these contact lenses [37]. Further details on the power profiles of the most current multifocal contact lenses for presbyopia correction can be found in previous publications: Plainis et al. [37], Montés-Micó et al. [38], Wagner et al. [39], and Kim et al. [40].

There are two main types of simultaneous image contact lens designs *concentric multifocal contact lenses* and *aspheric multifocal contact lenses.*

- Concentric multifocal contact lenses: these contact lens designs have a primary viewing zone in the center of the lens, which provides either distance or near power, surrounded by concentric rings of near or distance power, respectively (see Figure 1). These lenses are designed as near-center or distance-center and are classified as biconcentric or multiconcentric [41–43].
- Aspheric multifocal contact lenses: these contact lenses designs are based on aspheric designs fitted by conics, allowing the manipulation of the spherical aberration to modify the depth of focus. These designs comprise a power gradient that changes radially across the lens, most frequently in a radially symmetric fashion [44].

Unlike the discrete segmented rings of distance and near refractive power surrounding the center of the lens in the concentric designs, the aspheric designs show gradual changes in power from the center (center-distance or center-near) to the periphery of the lens (see Figure 1).

### 3.3. Characterization of the Simultaneous Image Contact Lens Design

Recent studies have published the designed phase patterns of different multifocal contact lens models (e.g., Charman described in its review article the power profile of the Purevision and Acuvue Oasys along a radius of 3.0 and 3.5 mm, respectively, of nominally 0.00 D distance power [32]). This information is essential to develop realistic individual simulations on model eyes, understand the multifocal performance of different distributions of near/far zones across the pupil, and interpret the visual outcomes [37, 38, 43, 45].

**Figure 2:**
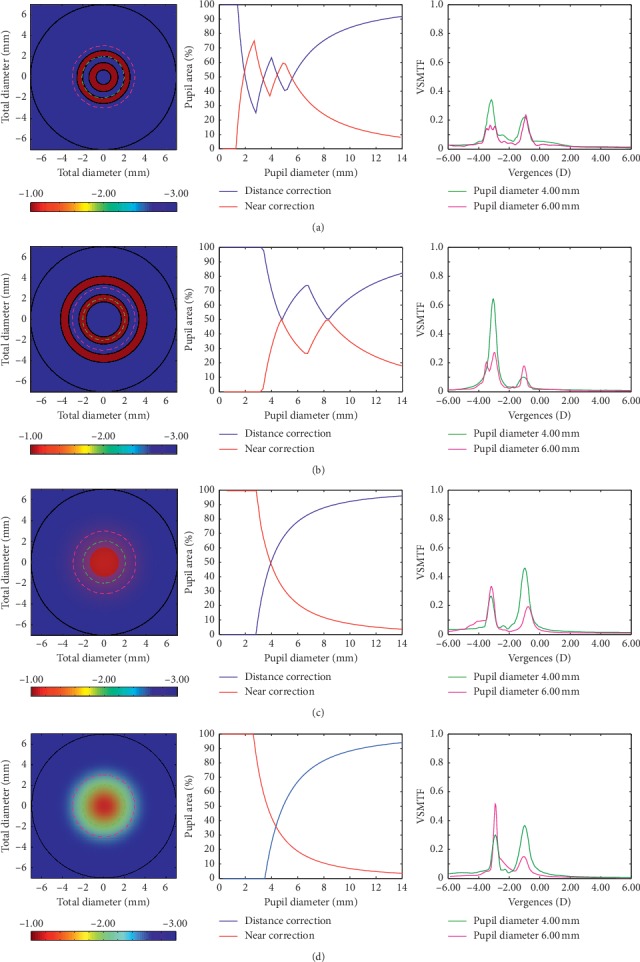
Illustration of the power maps (left), the proportion of the total pupil area covered by the distance and near correction as a function of the pupil diameter (center), and the through-focus Visual Strehl for pupil diameters of 4 mm and 6 mm (right) of different simultaneous image multifocal and bifocal contact lenses: (a) Acuvue Oasys for presbyopia, (b) Dual Focus, (c) PV: Purevision and (d) Airoptix (redrawn from Plainis et al. [37]). Profiles are designed to provide a distance correction power of −3.00 and an addition power of +2.00 diopters resulting in −1.00 of near correction power.

To date, there are different commercial devices that measure objectively the contact lens power profile and power maps following the specifications of ISO 18369-2:2013 (Ophthalmic optics—Contact lenses—Part 2: tolerances) [46] and ISO 18369-3:2017 (Ophthalmic optics—Contact lenses—Part 3: measurement methods) [47]: *ConTest II* (Rotlex, Israel), which uses a Moiré fringe method; *Visionix 2001* (Visionix Ltd, Jerusalem, Israel) [43] and *SHSOphthalmic* (Optocraft GmbH, Erlangen, Germany) [39], which are based on Hartmann-Shack technology; and *NIMO TR1504* (Lambda-X, Nivelles, Belgium), based on a deflectometry technique and the combination of the Schlieren principle with a phase-shifting method [38, 45]. In Figure 2, we illustrate the power maps (left), the proportion of the total pupil area covered by the distance and near correction as a function of the pupil diameter (center), and the through-focus Visual Strehl (right) for four different soft multifocal contact lenses (A: Acuvue Oasys for presbyopia, medium addition; B: Dual Focus for myopia progression control; C: Purevision Multifocal, high addition; D: Airoptix, as they exemplify different design concepts, are widely used in the clinical practice and information exist about their visual performance in the literature [37]). Two of these lenses (A and B) have a multizone design with central-far design, while C and D are center-near designs. Acuvue Oasys (A) differs from Dual Focus (B) in the size of the zones, especially in the central annular. The consequence is an improvement for distance vision, especially with small pupil sizes (around 2 mm). For higher pupil diameters, the area of the pupil is covered equally by far and near vision corrections, ensuring reasonable contrast for both far and near images. Dual Focus (B) provides a clear dominance for far focus with different pupil diameters. Purevision Multifocal (C) and Airoptix (D) become strongly biased towards distance correction as the pupil diameter increase. The design of these multifocal contact lenses (C and D) differs in the transition zone between near and distance vision.

## 4. Simultaneous Image

**Figure 3:**
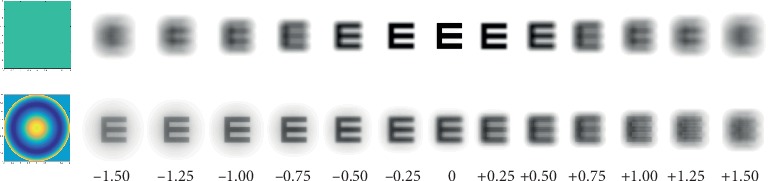
Illustration of the phase pattern and through-focus theoretical simulations of the Snellen E-letter of 30 arc-min for 4 mm pupil diameter (from −1.5 to +1.5). Top: diffraction-limited eye; bottom: aspheric-based design (spherical aberration: +0.25 *μ*m).

In simultaneous image contact lenses, the resultant image is a sharp image (in-focus) superimposed on a blurred background from the out-of-focus images, and it is expected that patients are able to progressively adapt to this simultaneous image situation [48]. However, this blurred background is sometimes described as ghost images or halo by patients.  Figure 3 illustrates the Snellen E-letter for a theoretical diffraction-limited eye (top) and an aspheric-based design with +0.25 spherical aberration (bottom) from −1.75 to +1.75 diopters (D). This figure exemplifies the challenges potentially faced by subjects when viewing through multifocal simultaneous vision contact lenses (Figure 3 bottom). Beyond the function for which the lens is designed, either enhancing the depth of focus or halting myopia progression, the device has to provide functional visual acuity at different target distances either without accommodation or with minimal residual accommodation in the older presbyopic eye or couple with the subjects accommodation in the case of the younger eye in myopia control devices.

**Figure 4:**
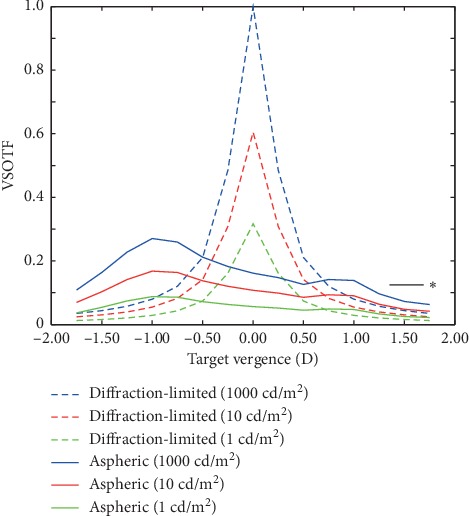
Through-focus Visual Strehl for the theoretical diffraction-limited eye (dashed) and the aspheric design (solid) for 4 mm pupil diameter and different light level conditions: 1000 cd/m^2^, 10 cd/m^2^, and 1 cd/m^2^. ^*∗*^ Threshold for acceptable vision.

Multifocal contact lenses relay strongly on centration in the pupil and pupil size variations with luminance and/or aging (note that only rays of light of the multifocal pattern transmitted through the pupil are relevant to the visual performance).  Figure 4 illustrates the through-focus Visual Strehl of the theoretical diffraction-limited eye and the aspheric-based design for different environmental light levels (from high-photopic 1000 cd/m^2^ to mesopic 1 cd/m^2^). The theoretical performance of the aspheric-based design shows a depth of focus of 2.5 D under high-photopic conditions (for 4 mm pupil diameter) but is limited to 1.0 D for low-photopic conditions and under the threshold in mesopic environmental light levels.

In addition to these limitations, multifocality and blur tolerance vary substantially across individuals due to ocular aberrations and neural adaptation. Therefore, understanding the coupling effect between the contact lens design, ocular optics, and visual adaptation is essential to explain the mechanism of action of a specific multifocal design for presbyopia and myopia applications.

### 4.1. Evaluation of the Visual Performance in Simultaneous Image Designs

Most clinical studies with multifocal contact lenses are limited to reports of through-focus visual acuity and contrast sensitivity, generally aiming at a depth of focus analysis and the improvement in near vision without compromising distance visual acuity. Aberrometry is an important clinical tool for objective evaluation of the image quality and visual performance prediction; however, due to the coupling of the phase of concentric multifocal designs there are some technical difficulties in the wavefront reconstruction of current sensors (true ocular aberrations and the power distribution in the pupil area), requiring an accurate reconstruction method for a proper combination of the wavefront slopes estimated at far and near distances [49]. Recently, theoretical visual simulations in eye models with multifocal designs and experimental visual simulators have shown the theoretical and real visual performance of different lens designs.

Computational models revealed that the multifocal benefit varied with the number of multifocal zones, showing that multiple refractive zone concentric rings (up to 3-4) were more robust in expanding the depth of focus for different pupil sizes than two-zone designs [50]. However, unlike theoretical models, experimental visual simulators incorporate both ocular optics and neural factors, showing visual testing of different multifocal patterns and offering patients a direct visual experience before fitting a specific multifocal contact lens design. These simulators are based on adaptive optics elements (deformable mirrors or spatial light modulators [51–53]) or temporal multiplexing (e.g., SimVis technology [54–56]) and work by projecting the theoretical multifocal pattern design onto the patient´s pupil plane, allowing us to evaluate the effect of different distance-near pupillary distribution and to test directly the visual performance. Recent studies have demonstrated that the through-focus visual performance with the same multifocal pattern varied across individuals, indicating that the specific performance of the design is highly patient-specific since not all patients tolerate well the out-of-focus image components in simultaneous vision [55]. de Gracia et al. [50] showed that the amount of near addition affected visual acuity differently, with the largest decrease for intermediate additions (around 2D). In addition, Radhakrishnan et al. [57] demonstrated that the perceived visual quality under simultaneous vision is affected by both the near addition magnitude and the distance-near energy ratio, showing maximal perceptual degradation at around 0.5D additions. Dorronsoro et al. [48, 55] found that bifocal rotationally asymmetric designs outperform other designs in real subjects. Different studies have also shown that there is an adaptation to the amount and orientation of blur caused by high-order aberrations [58, 59]. Interestingly, different patients preferred different orientations of the multifocal pattern (specifically, for angular designs [60, 61]) and this subjective orientation preference was predicted by ocular aberrations [48].

## 5. Patient Selection Criteria

This section intends to discuss the ocular factors that affect the performance of multifocal devices. The frequency of selecting a multifocal correction for presbyopia correction or in myopia progression control, as well as the number of designs commercially available, is rapidly increasing. However, the adaptation of multifocal contact lenses is still challenging for patients and practitioners. The problem is more complicated than coupling the multifocal design of the lens and an average value of spherical aberration for the eye (e.g., +0.25 *μ*m), as one needs to consider other critical factors for considering the optimum optical design for presbyopia or myopia application: *pupil diameter* (especially, variations with accommodation, aging, and lighting levels); *ocular changes with accommodation and aging* (in particular, the magnitude and sign of astigmatism and/or spherical aberration); the *on-eye performance* (since depending on the ocular aberrations the lens design could add other ocular aberrations or subtract them); and the *tear film dynamics* (with aging there is a generalized decrease of tear production and stability).

### 5.1. Pupil Diameter

Winn et al. [62] investigated the variation in pupil size over a large range of age and luminance levels, showing that the pupil size becomes smaller in an almost linear manner with increasing age (see Figure 16 at ref. [32]). The typical pupil diameter for a luminance level of approximately 220 cd/m^2^ in subjects between 20 and 29 years is around 5.5 mm, in subjects between 50 and 59 years old is around 4.5 mm, and in subjects between 70 and 79 years is around 3.5 mm. The average presbyopic pupil size for distance vision is below 5.00 mm in diameter under any light conditions, and a pupil diameter higher than 6.00 mm would be expected with younger presbyopes and under low lighting conditions. As mentioned above, the pupil diameter changes with the accommodation, so that the near pupil is smaller than the distance pupil. This fact is more relevant in younger subjects than in presbyopic subjects where the ability to accommodate is reduced. The reduced pupil diameter has the potential disadvantage of leading to lower retinal illuminance that affects the visual performance under low levels of illumination. However, smaller pupils have the advantage of producing an increasing depth of focus and better visual performance at distance because the peripheral less-focused light is excluded. Furthermore, although the high-order aberrations increase with age, its impact is attenuated when the pupil size decreases. In addition to the effect of pupil size on light transmission, the pupil size also may influence the effectiveness of the photoreceptor function due to the directional sensitivity of the photoreceptors (Stiles-Crawford effect). Despite its retinal origin, it may be regarded as effectively because of apodization at the pupil plane; so the rays passing through the pupil periphery have lower transmission in comparison to the central pupil. The potential benefits of the Stiles-Crawford effect are greatest with large pupils, while pupils smaller than 4 mm tend to minimize this effect affecting the retinal image quality significantly. So, the reintroduction of pupil transmission apodization is considered as an option to improve the through-focus retinal image quality [63–66]. Zheleznyak et al. [67] demonstrated that the pupil's periphery contains near addition power for positive spherical aberration, similar to center-distance designs. As a result, presbyopic eyes with negative spherical aberration improved with pupil transmission apodization.

### 5.2. Ocular Aberrations

Because of structural changes in the crystalline lens (shape, position, and refractive index) that occur during accommodation, wave aberrations are expected to change. Spherical aberration has been reported to shift towards negative values, and different studies also showed changes in coma, trefoil, and astigmatism, but the direction of the change was variable [68–73]. With aging, the optical performance of the eye also changes. Due to the disruption of the compensatory effect between the anterior cornea and the internal aberrations, there is an increase in high-order aberrations. In particular, the spherical aberration and horizontal coma tend to increase in older eyes [74]. Tabernero et al. [75] showed that the RMS of the higher-order ocular and corneal aberrations increased with age at a rate of 0.0032 *μ*m/year and 0.0015 *μ*m/year, respectively. In this study, the authors did not observe changes in the optical alignment with age (i.e., the angle kappa remains stable), assuming, therefore, that variations in the crystalline lens shape with age might explain most of the increment of ocular aberrations. Interestingly, it has been also demonstrated that the optical quality could be improved by adding certain amounts of spherical aberration to a given level of defocus, as well as specific amounts of astigmatism and coma can interact favorably to increase the depth of focus while minimizing the decrease of visual acuity [76, 77]. Therefore, the aberrations of individual eyes will determine the effectiveness of a multifocal correction and the achieved depth of focus.

### 5.3. Off-Axis Ocular Aberrations

A comparison between refractive groups shows that myopic eyes have more relative peripheral defocus as well as a prolate retinal shape than emmetropic and hypermetropic eyes. However, substantial differences in relative peripheral refraction for different degrees of myopia appear at high eccentricities of the visual field. The horizontal meridian is more myopic than the vertical meridian. The largest off-axis optical aberrations are represented by oblique astigmatism, which is induced by the oblique angle, and coma, showing little difference between refractive groups. The spherical aberration is more positive for hyperopes than for myopes and emmetropes [78–80].

### 5.4. On-Eye Contact Lens Performance

When a contact lens is placed on the eye, there is an interaction between the lens design and the patients' native aberrations. One of the most common options used to expand the depth of focus is by modulating the magnitude of the spherical aberration. Multifocal center-near designs commonly have a negative spherical aberration; however, there is wide individual variability in the spherical aberration coefficient across the population. Therefore, it is possible to find similar values of ocular spherical aberrations but opposite in sign in comparison to the lens design, reducing or cancelling the expected depth of focus [81–85]. Also, the on-eye performance of the contact lens may induce aberrations due to decentration [86]. As multifocal contact lens designs become more complex, centration is more critical. Decentration is due to lens flexure or fitting results in an induction of astigmatism and coma, with this induction being proportional to the amount of decentration. Likewise, decentration of a multifocal design with a higher magnitude of spherical aberration will produce higher magnitude of inducing astigmatism and coma; this could be of practical significance since many contact lenses wearers have their astigmatism uncorrected. The connection between spherical aberration and coma and the possibility of balancing coma by modulation of aspheric designs are recognized in the classic Seidel aberration theory; so, luckily to date, there are some strategies that modulate the optical surfaces to decrease the impact of decentration (e.g., aspheric balance curve) [87]. Furthermore, with binocular viewing multifocal concentric designs showed temporal decentration, supporting the strategy of asymmetrical concentric multifocal design to coincide with the line of sight [88–90].

### 5.5. Tear Film

Changes in the tear fluid dynamics can induce changes in high-order aberrations [91–94]. Koh et al. [91, 95] demonstrated that during dynamic aberrometry (10 seconds after blinking), most of the clinically normal subjects showed fluctuations in the high-order aberration pattern, with these fluctuations being higher in patients with tear film instability and ocular surface damage.

### 5.6. Accommodation

The interactions between the multifocal designs and the subject´s accommodative response should be considered to evaluate the visual performance in the myopia control application.

## 6. Performance of Contact Lenses for Presbyopia Correction

Evaluating the performance of contact lenses for presbyopia correction requires different levels of analysis, including the assessment of visual acuity at different distances/vergences, contrast sensitivity function under different lighting levels, stereoacuity, and the occurrence of subjective complains related to dysphotopsia [96]. For clarity, the binocular visual performance is presented in this section. The monocular performance is usually worse and, in some cases, asymmetric between dominant and nondominant eye, with possible implications in stereoacuity [97].

### 6.1. Visual Acuity

High and low contrast LogMAR visual acuity has become the standard for clinical visual performance assessment during the last ten years (see Table 1). The results from Fernandes et al. [97] showed better high contrast visual acuity (HCDVA) compared to previous studies with Proclear multifocal [99] while high contrast near visual acuity (HCNVA) was comparable to previous results reported by Ferrer-Blasco and Madrid-Costa [106] and slightly better than monovision fitting with the single vision Proclear lens. Gupta et al. [99] compared a multifocal contact lens (Purevision multifocal) against monovision and showed a slightly poorer performance for monovision in terms of distance visual acuity as in our study. The present sample is very similar to that study in terms of sample size and procedures. Similar results to those reported by Gupta et al. [99] and from Fernandes et al. [97] have been reported by Richdale et al. [98] for Monovision compared to multifocal soft contact lenses (SofLens Multifocal, B&L). Those authors also measured high and low contrast distance and near LogMAR visual acuity also presenting the values for spectacle correction (Baseline). Results from Fernandes et al. [97] were within ±1 line of their reported VA for all the experimental conditions except for Monovision under near low contrast visual acuity (LCNVA), which performed better than Multifocal lens in their study. Recently, several clinical studies also evaluated visual performance with different contact lenses [103–105, 107]. Bakaraju et al. [103] measured the high contrast visual acuity (HCVA) and the low contrast visual acuity (LCVA) for the Airoptix Aqua, the Acuvue Oasys and extended depth of focus (EDOF) contact lens. They found that the EDOF provided better intermediate and near visual performance, with no difference for distance vision in comparison with the other multifocal contact lens designs. In a different study, Diec et al. [107] investigated if the initial multifocal contact lens performance predicts short-term dispensing performance, but their results were not able to predict the short-term performance of a multifocal contact lens.

### 6.2. Contrast Sensitivity Function

Contrast sensitivity function has been recorded in different studies with different instruments, being a remarkable limitation due to the lack of comparability among them [108]. More recently, the Functional Acuity Contrast Test (F.A.C.T) housed on a Functional Visual Analyzer machine (StereoOptical Co. Inc., Chicago, IL) for spatial frequencies of 1.5, 3, 6, 12, and 18 cycles/degree has been increasingly used. This device allows a systematic control of distance of examination and luminance conditions and has proved to report comparable values to Vision Contrast Test System VCTS 6500 (Vistech Consultants, Dayton, OH) in the same study. A summary of different studies reporting binocular distance contrast sensitivity is presented in Table 2.

In a study conducted by Fernandes et al. [97], it was remarkable that in spite of the good vision that Monovision patients have at distance in the dominant eye, they do not perform better than Biofinity MF after 15 days of lens wear. Similar results have been reported for distance vision with Distance contrast sensitivity function (CSF) at 3 m using the VCTS 6500 by Gupta et al. [99] comparing a multifocal contact lens and Monovision.

Llorente-Guillemot et al. [101] and Madrid-Costa et al. [102] measured the contrast sensitivity under photopic as well as mesopic conditions and found an overall decay. The loss of sensitivity was in the range of 0.25 LogCS units for lower frequencies of 1.5 and 3 cpd and 0.05 to 0.10 for medium frequencies of 6 and 12 cpd. Interestingly, it was under the mesopic conditions where the lenses under comparison presented statistically significant differences. For example, Madrid-Costa et al. [102] did not find significant differences between Acuvue Oasys and Purevision under photopic conditions but did for mesopic conditions at 6, 12, and 18 cpd where the Purevision lens performed significantly better. Llorente-Guillemot et al. [101] showed that the presence of glare could decrease further the performance of Purevision multifocal compared to spectacle correction.

Different authors have also measured the CSF at near for presbyopic patients wearing contact lens correction for presbyopia, and these results are presented in Table 3. When compared with distance values of CS, some authors found similar values between distance and near as in the case of Llorente-Guillemot et al. [101] while others found systematically higher values for low and medium frequencies at distance while found higher CS values at the higher frequency (18 cpd) at near [99]. Madrid-Costa et al. [102] using the same measuring device, obtained much lower values of CS for the low and medium frequencies at near and similar values for the highest frequencies of 12 and 18 cpd. Differences in the control of the near distance, ambient illumination, age of the patients, and the impact of different lens designs used, might explain such a diversity of trends when trying to compare distance and near values of CS among studies.

### 6.3. Steroacuity

Stereoacuity is relevant in presbyopia correction with contact lenses because some modalities, as monovision, affect the ability of both eyes to work together in an effective way to the highest level of binocularity and stereoscopic perception. In a study conducted by Fernandes et al. [97] in 20 presbyopes wearing Biofinity single vision lenses for monovision and Biofinity Multifocal, stereoacuity was obtained with the Stereo Fly SO-001 (StereoOptical Co, Inc., Chicago, IL). There were statistically significant differences in stereopsis between both modalities being worse for Monovision, as expected (*p*=0.002). Furthermore, values for this parameter in the Monovision group were quite scattered with patients showing much worse outcomes than others. Such differences between groups remained even after 15 days of adaptation. Values for multifocal lenses after 15 days were very similar to those obtained by previous authors with other multifocal lenses using other tests [106] and with Proclear Multifocal using the same test [109]. The main results of several recent studies are summarized in Table 4.

Overall, it can be observed that all simultaneous image multifocal and bifocal soft contact lenses provide a good level of stereoacuity while monovision significantly impairs this function. Moreover, the effect of monovision in stereoacuity seems to remain unchanged after 15 days wearing the modality, which suggests that if stereoacuity improves over time with monovision, this is not likely to happen in the short term.

### 6.4. Through-Focus Performance

Through-focus performance is reported in the so-called defocus curves, which provide information on the visual performance of the presbyopic patient at different vergence distances. While used extensively in clinical research related to surgical solutions for presbyopia [110], it has not been until recently that these metrics have been more intensively applied to the assessment of multifocal contact lenses.

We have to differentiate the through-focus performance of through-focus curves from the depth of focus (DoF), which is the ability of the eye to see objects in a relatively wide range of vergences or distances without changing the accommodation. This phenomenon has been extensively reviewed by Wang and Ciuffreda [111] and their work provides relevant information that might also apply in the context of multifocal contact lens performance because, if the DoF changes with age, pupil size, or other factors associated with the ageing process of the human eye, this might also affect the performance of the patients and this might limit our ability to discriminate which part of the improvement effect with a certain contact lens is associated with the optics of the lens itself or to the DoF of the patient. According to the summary, they provide in Table 5 the average DoF of the eye ranges from 0.13 to 0.5 D approximately. Their summary of information also shows that for the majority of studies dealing with different variables, DoF increases with ageing and is better for smaller pupil sizes. Both factors will certainly play a role in the performance of presbyopes with multifocal contact lenses and highlight the importance of the pupil size in multifocal contact lens performance.

The first references to the analysis of defocus curves in contact lenses are found in Bradley and coauthor's work, back in the 1990s [115]. They evaluated the through-focus performance in two subjects wearing a single vision, a 2-zone bifocal, and a diffractive bifocal contact lens by assessing the contrast sensitivity for a 6/9 (20/30) visual acuity letter over a range of +2 to −4 D of vergence, in 0.5 D steps. Their results showed an extension of the depth of focus with the bifocal refractive and diffractive contact lenses at the expense of an overall drop in contrast sensitivity at distance compared with the single vision lens. In one subject, the depth of focus was expanded from distance to a vergence of 2.5 D if a cut-off point is set at 0.6 log CS values [115].

Gupta et al. [99] showed that the defocus focus performance in early presbyopes between 45 and 55 years of age was similar between monovision and multifocal aspheric center-near lenses. Their results showed an average LogMAR visual acuity for monovision and multifocal lenses of 0.00 and 0.05 at distance, 0.05 and 0.05 at intermediate vision at 66.67 cm, and an average 0.32 and 0.40 at 33 cm (−3 diopters of vergence), respectively. Madrid-Costa et al. [102] evaluated the performance of two different refractive multifocal soft contact lenses with an aspheric center-near design (Purevision Multifocal) and a zonal concentric design (Acuvue Oasys for Presbyopia). Both lenses performed similarly for distance and intermediate distances, but the Purevision lens performed slightly better by half a line of visual acuity for near distance. Table 5 shows the results of different studies evaluating the defocus curves with different multifocal contact lenses.

**Figure 5:**
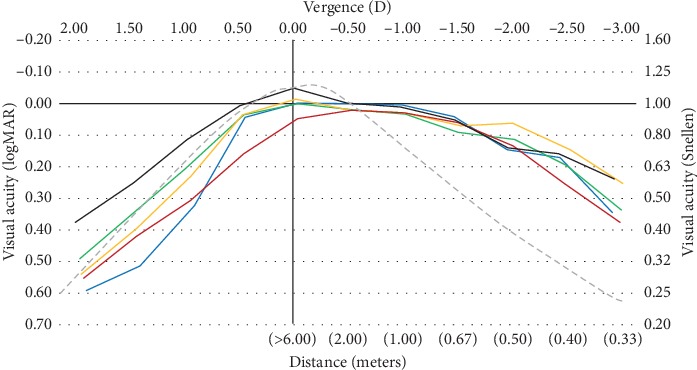
Reconstruction of the binocular defocus curves drawn at the same scale from different studies: red line: 20 subjects (49–67 years of age) fitted with Softlens multifocal (Gupta et al. [99]). Blue line: 20 subjects (age: 45–63 yrs) Proclear Toric Multifocal (Madrid-Costa et al. [113]). Orange line: 20 subjects (age: 42–48 yrs) Acuvue Oasys (Madrid-Costa et al. [102]). Green line: 20 subjects (age: 42–48 yrs) Acuvue Oasys presbyopia (Madrid-Costa et al. [102]). Black line: 38 subjects (age: 48–62 yrs) Proclear Multifocal (Garcia-Lázaro et al. [116]). Dashed grey line: expected performance for fully presbyopic eyes (unpublished data from CEORLab-UMinho).

According to the power distribution in multifocal contact lenses [37, 38] along with the computational predictions (see Figures 2 and 3), different multifocal contact lenses in the market should render significantly different performance. However, as seen in different studies, the clinically recorded through-focus curves are very similar (Figure 5). Interestingly while all the lenses give the same result for negative vergences, positive vergences render different results between different lenses. This might be related to the spherical aberration of the contact lenses and the ability to couple constructively with the positive spherical defocus to sustain or degrade vision as defocus increases. The common behavior for negative vergences might be the result of statistical regression to the mean for each vergence such that despite the different performances of different patients, the average behavior is very similar when compared between samples of different studies. Thought with some differences due to the ability to control several variables such as pupil size and aberration structure of synthetic eyes, similar results were found by Faria-Ribeiro et al. [117] when evaluating the through-focus performance of different contact lens designs. The same study confirms the variability of performance with varying pupil sizes and over and less than average spherical aberration. Altogether this confounding results point to the need to better match the lens design to the pupil size and remaining characteristics of the patient as described in the previous section regarding patient selection criteria. In the near future it should be possible to develop more sophisticated fitting algorithms that take into account all these variables.

## 7. Performance of Contact Lenses for Myopia Control

Considering the intended treatment, myopia control contact lenses have to prove efficacy in the reduction of axial elongation besides providing appropriate visual performance and safety. Till recently, the use of soft contact lenses for myopia control has been done off-label, and few have been subject to clinical trials to evaluate the longer-term efficacy of these devices. Those include two multifocal soft contact lenses for presbyopia correction used successfully to reduce myopia progression. Aller et al. [118] obtained a reduction of 72% in axial elongation in pediatric eyes wearing Acuvue Bifocal contact lens. Walline et al. [2] obtained a 29% reduction in axial elongation with Proclear Multifocal center-distance design in a pediatric population. Over the last 10 years, at least 5 different contact lenses specifically designed for myopia control in children have been subject to clinical trials. Those include peripheral gradient contact lenses that emulate the peripheral convergent power induced by orthokeratology [119], soft contact lenses that induce negative spherical aberration with the intended effect of improving accommodative response in myopic children [120], bifocal/dual-focus contact lenses with larger central zone devoted to distance vision [42, 121–123], and extended depth of focus contact lenses with alternating areas of positive and negative power modulated by inducing primary and secondary spherical aberration on the front surface of the contact lens ([119]; see also medium-add power design in Bakaraju et al. [103, 124] for further information about lens design).  Table 6 presents a summary of some relevant aspects to be considered when evaluating the performance of bifocal and multifocal contact lenses for myopia control in children.

### 7.1. Visual Acuity and Dysphotopsia

Since a pediatric patient has generally full accommodation capability, near vision is not usually a concern in visual evaluation. However, the ghosting induced by some contact lens designs used for myopia control requires that near vision needs to be assessed. Other more sophisticated modes to evaluate vision should be used in the future as myopia control devices can induce some degree of dysphotopsia, particularly under dim lighting conditions [85, 127]. These complaints could worsen as the children evolve into young-adults, and they are exposed to situations where these complaints might be more noticeable with bifocal and multifocal contact lenses or even orthokeratology [128] such as night driving. Those results in the context of clinical trials as well as experimental studies conducted recently with different lens prototypes [129] reveal that for the pupil size of younger subjects, distance visual performance could not be compromised as measured with visual acuity charts as long as the lens preserves a significant proportion of the optic zone devoted to distance vision focus. These findings are compatible with the simulations presented in Figure 2(b) such that better distance performance is warranted for larger pupil sizes with the lens specifically designed for myopia control in children (dual-focus, MiSight, Coopervision).

### 7.2. Accommodation Function

Few studies have evaluated the accommodation and binocular vision balance in children undergoing myopia control treatments. In the context of the Cambridge Antimyopia Study, though improvements in accommodative efficiency were observed with soft contact lenses inducing negative spherical aberration, these devices were not successful in retarding axial elongation in teenagers [120]. Other studies reporting such results generally agreed that no significant changes are observed with peripheral gradient contact lenses [11], concentric dual-focus [42, 122], and defocus incorporated soft contact lens [125]. This is consistent with the computational calculations of Faria-Ribeiro and González-Méijome [130], who found no visual advantage in using the near focus to see closer to the add power of the lens with the current designs of dual-focus lens. This is consistent with the wider area of distance vision in the contact lens with proximal miosis. Instead, some benefit in using the near focus of the lens if the central zone of the lens dedicated to distance vision was narrower. However, such undesirable effect, as underaccommodation at near vision, would result in hyperopic defocus for light refracting through the distance zone under such accommodation inhibition at near.

### 7.3. Ocular Length Retardation

Retardation of eye growth has been the main outcome in most clinical trials related to the use of contact lenses for myopia control. The effect varies from nearly 30% in some studies to over 70% in others [2, 118]. In some instances, the same device renders quite different efficacy results. However, we have to bear in mind that the current approach to myopia control with bifocal and multifocal lenses uses unique “treatment” parameters for the same device, and therefore the same “dose” is applied to all patients. As discussed for multifocal contact lenses for presbyopia and their similar performance for defocus curves, better patient-to-device selection algorithms could provide better results in the future. However, this will require a better understanding of the mechanisms governing the myopization process in young children and the mechanisms of action of optical devices that are able to control the ocular growth.

### 7.4. Adverse Events

Adverse events have been rarely found in the context of clinical trials involving pediatric populations wearing contact lenses for the purpose of myopia control for periods from 1 to 3 years. The attrition of patients to the study varies from over 80% in some studies to less than 60% in others [131]. However, this seems not to be related to the performance or safety of the contact lenses and most studies show that those discontinuing their participation do so for other aspects not related to adverse events.

## 8. Conclusions

Current bifocal and multifocal contact lens designs for presbyopia correction and myopia progression control are focused on providing a robust distance and near visual performance over a wide range of pupil sizes. However, considering the different purposes (presbyopia vs. myopia), ocular characteristics (young vs. adults), and neural adaptation, the bifocal and multifocal design of the contact lenses should evolve in different directions considering the significant difference in pupil sizes and the aberrometric profile of the potential candidates for presbyopia correction or myopia control. Presbyopia correction is now available over a diverse range of material platforms including soft, hybrid, corneal, and scleral rigid gas permeable contact lenses. Besides orthokeratology, performance with corneal rigid gas permeable contact lenses, myopia control evolves mainly in the soft contact lens materials with several lenses undergoing long-term clinical trials (2 or more years). While presbyopia correction with contact lenses accounts for up to 25 to 35% of the contact lens fittings in several countries [5], myopia control contact lens fittings are still limited to 2 to 5% of the contact lenses fitted [6]. Considering the positive results with the contact lenses evaluated in the studies summarized in this review, the demographic trends and the increase in myopia among the younger, it is expected that both fields of contact lens application experience an expansion in number and diversity of devices being produced, subjected to clinical trials and launched to the market over the next decade. This trend might be more significant in the myopia control field considering the yet low penetration and the fact that contact lenses offer a nonpharmacological, minimally invasive, and well-accepted form of treatment. On the other side, the presbyopic correction might increase more moderately as it shares a significant market with the surgical interventions, and spectacle correction will probably continue being the dominant option for the next years. It will be interesting to follow these trends to understand where the next years take the contact lens field, with these two applications being at the forefront of the evolution requiring more effective designs.

## Figures and Tables

**Table 1:** Summary of results of recent studies evaluating the photopic binocular high and/or low contrast visual acuity at distance (4 to 6 m) and near (33 to 40 cm) in presbyopic patients fitted with simultaneous image contact lenses. Visual acuity is expressed in LogMAR units.

Author (year)	Lens type/fitting	n (Rx) (age)	Distance high contrast (LogMAR)	Distance low contrast (LogMAR)	Near high contrast (LogMAR)	Near low contrast (LogMAR)
Richdale et al. (2006) [98]	Monovision SofLens 59 MF	38 (−0.81 ± 0.10) (50.11 ± 4.70)	−0.10 ± 0.10 −0.12 ± 0.09	0.08 ± 0.15 0.08 ± 0.15	−0.03 ± 0.09 0.01 ± 0.12	0.14 ± 0.10 0.21 ± 0.14

Gupta et al. (2009) [99]	Monovision Purevision MF	20 (−1.42 ± 2.87) (55.0 ± 5.1)	−0.01 ± 0.07 0.05 ± 0.08		0.11 ± 0.11 0.21 ± 0.13	

García-Lázaro et al. (2012) [100]	Monovision Pinhole	22 (0.11 ± 0.12) (57.3 ± 5.8)	0.00 ± 0.09 0.02 ± 0.04	0.13 ± 0.12 0.16 ± 0.06	0.08 ± 0.16 0.40 ± 0.19	

Llorente-Guillemot et al. (2012) [101]	Spectacles Purevision MF	20 (−1.42 ± 2.87) (53.2 ± 5.3)	−0.05 ± 0.07 −0.01 ± 0.03	0.10 ± 0.06 0.18 ± 0.05	−0.08 ± 0.06 −0.02 ± 0.05	

Madrid-Costa et al. (2013) [102]	Purevision MF Oasys MF	20 (+0.35 ± 1.78) (45.1 ± 2.3)	0.00 ± 0.08 0.01 ± 0.08	0.11 ± 0.09 0.20 ± 0.58	0.15 ± 0.08 0.20 ± 0.05	

Fernandes et al. (2013) [97]	Monovision Biofinity MF	20 (−0.91 ± 2.25) 48.7 ± 3.3	−0.08 ± 0.09 −0.09 ± 0.08	0.11 ± 0.08 0.11 ± 0.06	0.05 ± 0.10 0.04 ± 0.07	0.23 ± 0.12 0.21 ± 0.09

Bakaraju et al. (2018) [103]	Airoptix Oasys MF EDOF	43 (−0.65 ± 0.88) (53 ± 5)	−0.07 ± 0.08 −0.06 ± 0.08 −0.07 ± 0.06	0.22 ± 0.10 0.27 ± 0.09 0.27 ± 0.10	0.13 ± 0.13 0.12 ± 0.11 0.10 ± 0.11	

Sha et al. (2016) [104]	Airoptix Oasys MF	42 (−0.35 ± 0.80) (58 ± 6)	−0.04 ± 0.06 −0.02 ± 0.09	0.28 ± 0.08 0.31 ± 0.12	0.48 ± 0.20 0.52 ± 0.22	

Tilia et al. (2017) [105]	Airoptix EDOF	41 (−0.6 ± 0.70) (53 ± 6)	−0.06 ± 0.05 −0.06 ± 0.05	0.25 ± 0.10 0.24 ± 0.04	0.48 ± 0.22 0.42 ± 0.18	

**Table 2:** Summary of results of recent studies evaluating the photopic binocular distance CSF in presbyopic patients wearing contact lenses for presbyopia correction. Units are LogCS.

Author (year)	Lens type/fitting	*n* (Rx) (age)	LogCS (1.5 cpd)	Log CS (3 cpd)	Log CS (6 cpd)	Log CS (12 cpd)	Log CS (18 cpd)
Gupta et al. (2009) [99]	Monovision Purevision MF	20 (−1.42 ± 2.87) (55.0 ± 5.1)	1.75 1.75	1.89 1.93	1.77 1.74	1.33 1.12	0.68 0.65

García-Lázaro et al. (2012) [100]	Monovision Pinhole	22 (0.11 ± 0.12) (57.3 ± 5.8)	1.49 1.40	1.69 1.64	1.46 1.41	0.94 0.90	0.63 0.60

Llorente-Guillemot et al. (2012) [101]	Spectacles Purevision MF	20 (−1.42 ± 2.87) (53.2 ± 5.3)	1.51	1.76	1.69	1.28	0.67

Madrid-Costa et al. (2013) [102]	Purevision MF Oasys MF	20 (+0.35 ± 1.78) (45.1 ± 2.3)	1.63 1.54	1.73 1.73	1.35 1.33	1.09 1.07	0.7 0.67

Bakaraju et al. (2018) [103]	Airoptix Oasys MF EDOF	43 (−0.65 ± 0.88) (53 ± 5)			1.47 1.44 1.44	1.26 1.21 1.21	1.01 0.92 0.95

**Table 3:** Photopic binocular near contrast sensitivity function for different studies. See also Table 2 for comparison with distance outcomes for the same studies. Units are LogCS.

Author (year)	Lens type/fitting	*n* (Rx) (age)	Log CS (1.5 cpd)	Log CS (3 cpd)	Log CS (6 cpd)	Log CS (12 cpd)	Log CS (18 cpd)
Gupta et al. (2009) [99]	Monovision Purevision MF	20 (−1.42 ± 2.87) (55.0 ± 5.1)	1.62 1.58	1.73 1.73	1.60 1.53	1.19 1.10	0.80 0.70

García-Lázaro et al. (2012) [100]	Monovision Pinhole	22 (0.11 ± 0.12) (57.3 ± 5.8)	1.52 1.48	1.60 1.43	1.49 1.21	1.09 0.79	0.85 0.60

Llorente-Guillemot et al. (2012) [101]	Spectacles Purevision MF	20 (−1.42 ± 2.87) (53.2 ± 5.3)	1.54	1.62	1.63	1.21	0.60

Madrid-Costa et al. (2013) [102]	Purevision MF Oasys MF	20 (+0.35 ± 1.78) (45.1 ± 2.3)	1.37 1.30	1.59 1.54	1.24 1.12	1.05 0.96	0.67 0.60

**Table 4:** Summary of results of recent studies evaluating stereoacuity with different methods in presbyopic patients wearing simultaneous image multifocal and bifocal soft contact lenses. The unit of stereoacuity is seconds of arc (arcsec).

Author (year)	Lens type/fitting	*n* (Rx) (age)	Method (s)	Stereoacuity (arcsec)
Richdale et al. (2006) [98]	Monovision SofLens 59 MF	38 (−0.81 ± 0.10) (50.11 ± 4.70)	Randot Preschool stereoacuity test	205 ± 214 126 ± 137

Gupta et al. (2009) [99]	Monovision Purevision MF	20 (−1.42 ± 2.87) (55.0 ± 5.1)	TNO random dot stereogram test	273 ± 102 174 ± 95.2

García-Lázaro et al. (2012) [100]	Monovision Pinhole	22 (0.11 ± 0.12) (57.3 ± 5.8)	Howard-Dolman system	210 ± 49 221 ± 32

Fernandes et al. (2013) [97]	Monovision Biofinity MF	20 (−0.91 ± 2.25) 48.7 ± 3.3	Stereo Fly SO-001	105 ± 95 51 ± 67

Bakaraju et al. 2018 [103]	Airoptix Oasys MF EDOF	43 (−0.65 ± 0.88) (53 ± 5)	Stereo Fly test Circles	97 ± 129 74 ± 63 61 ± 37

Sha et al. (2016) [104]	Airoptix Oasys MF	42 (−0.35 ± 0.80) (58 ± 6)	Stereo Fly test Circles	148 ± 131 100 ± 84

Tilia et al. (2017) [105]	Airoptix EDOF	41 (−0.6 ± 0.70) (53 ± 6)	Stereo Fly test Circles	141 ± 114 98 ± 88

**Table 5:** Results from the defocus curves obtained with different contact lenses in different studies. The approximate values have been extracted from the graphs presented by the authors for 0.0 D of vergence (distance), 1.0 D (1 meter), 1.5 D (67 cm), 2.5 D (40 cm), and 3.0 D (33 cm). Units are presented in LogMAR values. Above the shaded row are presented baseline data for no lens situation. Note that Plainis et al.'s [90] study has been performed on young people under cycloplegia.

Author (year)	Lens type/fitting	*n* (Rx) (age)	VA 0.0 D (distance)	VA −1.0 D (1 meter)	VA −1.5 D (67 cm)	VA −2.5 D (40 cm)	VA −3.0 D (33 cm)
Kingston and Cox (2013) [112]	Baseline (no lens)	64 eyes presbyopes	0.00	0.05	0.20	0.45	0.60

Plainis et al. (2013) [90]	Naked eye Monocular 3 mm pupil 6 mm pupil	12 (−2.24 ± 2.12) (27 ± 5) cyclopleged	−0.10 −0.10	0.0 0.5	0.10 0.20	0.32 0.36	0.42 0.52

Plainis et al. (2013) [90]	Naked eye Binocular 3 mm pupil 6 mm pupil	12 (−2.24 ± 2.12) (27 ± 5) cyclopleged	−0.15 −0.15	0.0 0.0	0.18 0.22	0.30 0.32	0.48 0.52

Gupta et al. (2009) [99]	Monovision Purevision MF	20 (−1.42 ± 2.87) (55.0 ± 5.1)	0.0 0.05	0.02 0.04	0.05 0.05	0.18 0.24	0.32 0.40

Madrid-Costa et al. (2012) [113]	Proclear MF toric	20 (−0.51 ± 2.01) (50.4 ± 7.8)	0.0	0.02	0.05	0.18	0.35

García-Lázaro et al. (2012) [114]	Monovision Pinhole	22 (0.11 ± 0.12) (57.3 ± 5.8)	0.0 0.0	0.18 0.07	0.18 0.20	0.08 0.4	0.3 0.52

Plainis et al. (2013) [90]	Airoptix MF Binoc 3 mm LOW Binoc 3 mm MED Binoc 3 mm HIGH	12 (−2.24 ± 2.12) (27 ± 5) cyclopleged	−0.15 −0.05 −0.04	−0.05 −0.05 −0.05	0.04 −0.06 −0.06	0.24 0.10 0.02	0.32 0.22 0.12

Plainis et al. (2013) [90]	Airoptix MF Binoc 6 mm LOW Binoc 6 mm MED Binoc 6 mm HIGH	12 (−2.24 ± 2.12) (27 ± 5) cyclopleged	−0.10 −0.02 −0.02	−0.02 −0.04 −0.02	0.08 −0.02 −0.06	0.30 0.10 0.05	0.40 0.24 0.16

Madrid-Costa et al. (2013) [102]	Purevision MF Oasys MF	20 (+0.35 ± 1.78) (45.1 ± 2.3)	0.0 0.0	0.04 0.04	0.06 0.08	0.16 0.20	0.24 0.34

Bakaraju et al. 2018 [103]	Airoptix Oasys MF EDOF	43 (−0.65 ± 0.88) (53 ± 5)	−0.07 −0.06 −0.07		−0.03 0.00 −0.07		0.13 0.12 0.10

Tilia et al. (2017) [105]	Airoptix EDOF	41 (−0.6 ± 0.70) (53 ± 6)	−0.06 −0.06		0.13 0.12		0.48 0.42

**Table 6:** Outcomes from clinical trials involving the use of bifocal/dual-focus and multifocal (including peripheral gradient and extended depth of focus) contact lenses for myopia control.

Author (year)	Lens design (trial duration, moths)	*N*	Axial growth (%)^*∗*^	Binocular distance visual acuity	Binocular near visual acuity HC	Accomm.	Wearing time hours/day	Discont.	Adverse events
Anstice and Phillips (2011) [42]	DF (10 months)	T: 52 C: 56	0.10 mm 0.22 mm (−55%)	99.9 ± 3.5^*∗∗*^ 100 ± 2.9		No change	13.2 ± 2.8 11.9 ± 2.0	N.R	N.R

Allen et al. (2013) [120]	−SA (24)	T: 29 C: 30	0.15 0.16 (−6%)	N.R	N.R	Improves Acc flexibility	N.R	12/41 15/45	N.R

Walline et al. (2013) [2]	CDMF (24)	T: 27 C: 27	0.29 0.41 (−29%)	N.R	N.R	N.R	N.R	5/32 5/32	N.R

Lam et al. (2014) [125]	DISC (24)	T: 65 C: 63	0.25 0.36 (−31%)	N.R	N.R	N.R	6.5 ± 2.2 6.3 ± 1.7	46/111 47/110	N.R

Cheng et al. (2016) [126]	+SA (12)	T: 53 C: 59		0.06 ± 0.06 0.00 ± 0.08	N.R	N.R	N.R	53/64 50/63	N.R

Aller et al. (2016) [118]	CDBF (12)	T: 39 C: 40	0.05 0.24 (−79%)	N.R	N.R	N.R	N.R		N.R

Pauné et al. (2016) [11]	PG (24)	T: 19 C: 21	0.38 0.52 (−27%)	N.R	N.R	N.R	N.R	11/30 20/41	N.R

Ruiz-Pomeda et al. (2018) [127]	DF (24)	T: 41 C: 43	0.28 0.45 (−38%)	N.R	N.R	N.R	12.2 ± 1.8 11.8 ± 2.1	5/41 0/33	N.R

Sankaridurg et al. (2019) [119]	EDOF (24)	T: 43 C: 39	0.44 0.58 (−24%)	0.07	Visual clarity subjectively reported better than distance	N.R	N.R	28/73 28/78	N.R

Chamberlain et al. (2019) [123]	DF (36)	T: 52 C: 56	0.30 0.62 (−52%)	0.00 ± 0.10 N.R	−0.10 ± 0.08	N.R	13.7 ± 1.5 13.3 ± 1.5	12/65 14/70	No serious adverse events

^*∗*^Axial length growth: defined as the % of growth in the test group compared to the control group [(Δ*T* − Δ*C*)/Δ*C*]; negative value implies a benefit of the treatment. ^*∗∗*^ Visual Acuity Rating Scale (100 = 6/6). DISC: defocus incorporated contact lens, concentric refractive; EDoF: extended depth of focus, only Design III is considered–currently manufactured by mark'ennovy; PG: peripheral gradient; DF: bifocal concentric design with large central zone for distance vision; CDMF: center-distance multifocal for presbyopia; CDBF: center-distance bifocal for presbyopia; +SA: soft contact lens with the induction of positive spherical aberration; −SA: soft contact lens with the induction of negative spherical aberration; T: test device: C: control device; ΔT|ΔC: increment in treatment|control groups; HC: high contrast; LC: low contrast N.R: not reported; Accomm.: accommodation; Discont.: discontinuation.

## References

[1] Bennett E. S. (2008). Contact lens correction of presbyopia. *Clinical and Experimental Optometry*.

[2] Walline J. J., Greiner K. L., McVey M. E., Jones-Jordan L. A. (2013). Multifocal contact lens myopia control. *Optometry and Vision Science*.

[3] Hiraoka T., Kakita T., Okamoto F., Takahashi H., Oshika T. (2012). Long-term effect of overnight orthokeratology on axial length elongation in childhood myopia: a 5-year follow-up study. *Investigative Opthalmology & Visual Science*.

[4] Gifford P., Swarbrick H. A. (2013). Refractive changes from hyperopic orthokeratology monovision in presbyopes. *Optometry and Vision Science*.

[5] Morgan P. B., Efron N., Woods C. A., Santodomingo-Rubido J. (2019). International survey of orthokeratology contact lens fitting. *Contact Lens and Anterior Eye*.

[6] Efron N., Morgan P. B., Woods C. A., Santodomingo-Rubido J., Nichols J. J. (2020). International survey of contact lens fitting for myopia control in children. *Cont Lens Anterior Eye*.

[7] Papadatou E., Del Águila-Carrasco A. J., Esteve-Taboada J. J., Madrid-Costa D., Cerviño-Expósito A. (2017). Objective assessment of the effect of pupil size upon the power distribution of multifocal contact lenses. *International Journal of Ophthalmology*.

[8] Rosén R., Jaeken B., Lindskoog Petterson A., Artal P., Unsbo P., Lundström L. (2012). Evaluating the peripheral optical effect of multifocal contact lenses. *Ophthalmic and Physiological Optics*.

[9] Pérez-Prados R., Piñero D. P., Pérez-Cambrodí R. J., Madrid-Costa D. (2017). Soft multifocal simultaneous image contact lenses: a review. *Clinical and Experimental Optometry*.

[10] Pauné J., Morales H., Armengol J., Quevedo L., Faria-Ribeiro M., González-Méijome J. M. (2015). Myopia control with a novel peripheral gradient soft lens and orthokeratology: a 2-year clinical trial. *BioMed Research International*.

[11] Pauné J., Thivent S., Armengol J., Quevedo L., Faria-Ribeiro M., González-Méijome J. M. (2016). Changes in peripheral refraction, higher-order aberrations, and accommodative lag with a radial refractive gradient contact lens in young myopes. *Eye & Contact Lens: Science & Clinical Practice*.

[12] Sun Y., Xu F., Zhang T. (2015). Orthokeratology to control myopia progression: a meta-analysis. *PLoS One*.

[13] Huang J., Wen D., Wang Q. (2016). Efficacy comparison of 16 interventions for myopia control in children: a network meta-analysis. *Ophthalmology*.

[14] Li S.-M., Kang M.-T., Wu S.-S. (2016). Efficacy, safety and acceptability of orthokeratology on slowing axial elongation in myopic children by meta-analysis. *Current Eye Research*.

[15] Li S.-M., Kang M.-T., Wu S.-S. (2017). Studies using concentric ring bifocal and peripheral add multifocal contact lenses to slow myopia progression in school-aged children: a meta-analysis. *Ophthalmic and Physiological Optics*.

[16] Prousali E., Haidich A. B., Fontalis A., Ziakas N., Brazitikos P., Mataftsi A. (2019). Efficacy and safety of interventions to control myopia progression in children: an overview of systematic reviews and meta-analyses. *BMC Ophthalmology*.

[17] van der Worp E., Johns L., Barnett M. (2019). Scleral schism. *Contact Lens and Anterior Eye*.

[18] Rosenthal P., Croteau A. (2005). Fluid-ventilated, gas-permeable scleral contact lens is an effective option for managing severe ocular surface disease and many corneal disorders that would otherwise require penetrating keratoplasty. *Eye & Contact Lens: Science & Clinical Practice*.

[19] Alipour F., Kheirkhah A., Behrouz M. J. (2012). Use of mini scleral contact lenses in moderate to severe dry eye. *Contact Lens and Anterior Eye*.

[20] van der Worp E., Bornman D., Ferreira D. L., Faria-Ribeiro M., Garcia-Porta N., González-Meijome J. M. (2014). Modern scleral contact lenses: a review. *Contact Lens and Anterior Eye*.

[21] Schornack M. M. (2015). Scleral lenses: a literature review. *Eye & Contact Lens: Science & Clinical Practice*.

[22] Visser E.-S., Van der Linden B. J. J. J., Otten H. M., Van der Lelij A., Visser R. (2013). Medical applications and outcomes of bitangential scleral lenses. *Optometry and Vision Science*.

[23] Visser E.-S., Visser R., Van Lier H. J. J. (2006). Advantages of toric scleral lenses. *Optometry and Vision Science*.

[24] Sorbara L., Maram J., Fonn D., Woods C., Simpson T. (2010). Metrics of the normal cornea: anterior segment imaging with the Visante OCT. *Clinical and Experimental Optometry*.

[25] Sorbara L., Maram J., Mueller K. (2013). Use of the Visante OCT to measure the sagittal depth and scleral shape of keratoconus compared to normal corneae: pilot study. *Journal of Optometry*.

[26] Ritzmann M., Caroline P. J., Börret R., Korszen E. (2017). An analysis of anterior scleral shape and its role in the design and fitting of scleral contact lenses. *Contact Lens and Anterior Eye*.

[27] Macedo-de-Araújo R. J., van der Worp E., González-Méijome J. M. (2019). In vivo assessment of the anterior scleral contour assisted by automatic profilometry and changes in conjunctival shape after miniscleral contact lens fitting. *Journal of Optometry*.

[28] Piñero D. P., Martínez-Abad A., Soto-Negro R. (2019). Differences in corneo-scleral topographic profile between healthy and keratoconus corneas. *Contact Lens and Anterior Eye*.

[29] Lee J. C., Chiu G. B., Bach D., Bababeygy S. R., Irvine J., Heur M. (2013). Functional and visual improvement with prosthetic replacement of the ocular surface ecosystem scleral lenses for irregular corneas. *Cornea*.

[30] Nichols J. J., Fisher D. (2019). Contact lenses 2018. *Contact Lens Spectrum*.

[31] Barnett M. (2015). Multifocal scleral lenses 2015. *Contact Lens Spectrum*.

[32] Charman W. N. (2014). Developments in the correction of presbyopia I: spectacle and contact lenses. *Ophthalmic and Physiological Optics*.

[33] Morgan P. B., Efron N. (2009). Contact lens correction of presbyopia. *Contact Lens and Anterior Eye*.

[34] Bennett E. (2010). Innovations in gas permeable multifocal contact lenses. *Clinical Optometry*.

[35] International Organization for Standardization (ISO) (2006). *Ophtalmic Optics-Contact Lenses, Part 1: Vocabulary, Classification System and Recommendations for Labelling Specifications*.

[36] Toshida H., Takahashi K., Sado K., Kanai A., Murakami A. (2008). Bifocal contact lenses: history, types, characteristics, and actual state and problems. *Clinical Ophthalmology*.

[37] Plainis S., Atchison D. A., Charman W. N. (2013). Power profiles of multifocal contact lenses and their interpretation. *Optometry and Vision Science*.

[38] Montés-Micó R., Madrid-Costa D., Domínguez-Vicent A., Belda-Salmerón L., Ferrer-Blasco T. (2014). In vitro power profiles of multifocal simultaneous vision contact lenses. *Contact Lens and Anterior Eye*.

[39] Wagner S., Conrad F., Bakaraju R. C., Fedtke C., Ehrmann K., Holden B. A. (2015). Power profiles of single vision and multifocal soft contact lenses. *Contact Lens and Anterior Eye*.

[40] Kim E., Bakaraju R. C., Ehrmann K. (2016). Reliability of power profiles measured on NIMO TR1504 (Lambda-X) and effects of lens decentration for single vision, bifocal and multifocal contact lenses. *Journal of Optometry*.

[41] Guispets J., Arjona M., Pujol J., Vilaseca M., Cardona G. (2011). Task oriented visual satisfaction and wearing success with two different simultaneous vision multifocal soft contact lenses. *Journal of Optometry*.

[42] Anstice N. S., Phillips J. R. (2011). Effect of dual-focus soft contact lens wear on axial myopia progression in children. *Ophthalmology*.

[43] Ruiz-Alcocer J. (2017). Analysis of the power profile of a new soft contact lens for myopia progression. *Journal of Optometry*.

[44] Pérez-Escudero A., Dorronsoro C., Marcos S. (2010). Correlation between radius and asphericity in surfaces fitted by conics. *Journal of the Optical Society of America A*.

[45] Madrid-Costa D., Ruiz-Alcocer J., García-Lázaro S., Ferrer-Blasco T., Montés-Micó R. (2015). Optical power distribution of refractive and aspheric multifocal contact lenses: effect of pupil size. *Contact Lens and Anterior Eye*.

[46] International Organization for Standardization (ISO) (2017). *Ophtalmic Optics-Contact Lenses, Part 2: Tolerances*.

[47] International Organization for Standardization (ISO) (2017). *Ophtalmic Optics-Contact Lenses, Part 3: Measurement Methods*.

[48] Dorronsoro C., Radhakrishnan A., de Gracia P., Sawides L., Marcos S. (2016). Perceived image quality with simulated segmented bifocal corrections. *Biomedical Optics Express*.

[49] Akondi V., Pérez-Merino P., Martinez-Enriquez E. (2017). Evaluation of the true wavefront aberrations in eyes implanted with a rotationally asymmetric multifocal intraocular lens. *Journal of Refractive Surgery*.

[50] de Gracia P., Dorronsoro C., Marcos S. (2013). Multiple zone multifocal phase designs. *Optics Letters*.

[51] Fernández E. J., Prieto P. M., Artal P. (2009). Binocular adaptive optics visual simulator. *Optics Letters*.

[52] Canovas C., Manzanera S., Schwarz C. (2014). Binocular performance of IOL combinations studied with a visual simulator. *Investigative Ophthalmology & Visual Science*.

[53] Hervella L., Villegas E. A., Prieto P. M., Artal P. (2019). Assessment of subjective refraction with a clinical adaptive optics visual simulator. *Journal of Cataract & Refractive Surgery*.

[54] de Gracia P., Dorronsoro C., Sánchez-González Á., Sawides L., Marcos S. (2013). Experimental simulation of simultaneous vision. *Investigative Opthalmology & Visual Science*.

[55] Dorronsoro C., Radhakrishnan A., Alonso-Sanz J. R. (2016). Portable simultaneous vision device to simulate multifocal corrections. *Optica*.

[56] Vinas M., Benedi-Garcia C., Aissati S. (2019). Visual simulators replicate vision with multifocal lenses. *Scientific Reports*.

[57] Radhakrishnan A., Dorronsoro C., Sawides L., Marcos S. (2014). Short-term neural adaptation to simultaneous bifocal images. *PLoS One*.

[58] Artal P., Chen L., Fernández E. J., Singer B., Manzanera S., Williams D. R. (2004). Neural compensation for the eye’s optical aberrations. *Journal of Vision*.

[59] Sawides L., Dorronsoro C., Haun A. M., Peli E., Marcos S. (2013). Using pattern classification to measure adaptation to the orientation of high order aberrations. *PLoS One*.

[60] Vinas M., Dorronsoro C., Gonzalez V., Cortes D., Radhakrishnan A., Marcos S. (2017). Testing vision with angular and radial multifocal designs using adaptive optics. *Vision Research*.

[61] de Gracia P., Hartwig A. (2017). Optimal orientation for angularly segmented multifocal corrections. *Ophthalmic and Physiological Optics*.

[62] Winn B., Whitaker D., Elliott D. B., Phillips N. J. (1994). Factors affecting light-adapted pupil size in normal human subjects. *Investigative Opthalmology & Visual Science*.

[63] Stiles W., Crawford B. (1933). The luminous efficiency of rays entering the eye pupil at different points. *Proceedings of the Royal Society of London Series B*.

[64] Applegate R. A., Lakshminarayanan V. (1993). Parametric representation of Stiles-Crawford functions: normal variation of peak location and directionality. *Journal of the Optical Society of America A*.

[65] Zhang X., Ye M., Bradley A., Thibos L. (1999). Apodization by the Stiles-Crawford effect moderates the visual impact of retinal image defocus. *Journal of the Optical Society of America A*.

[66] Atchison D. A., Scott D. H., Strang N. C., Artal P. (2002). Influence of Stiles-Crawford apodization on visual acuity. *Journal of the Optical Society of America A*.

[67] Zheleznyak L., Jung H., Yoon G. (2014). Impact of pupil transmission apodization on presbyopic through-focus visual performance with spherical aberration. *Investigative Opthalmology & Visual Science*.

[68] Artal P., Guirao A. (1998). Contributions of the cornea and the lens to the aberrations of the human eye. *Optics Letters*.

[69] Chen L., Kruger P. B., Hofer H., Singer B., Williams D. R. (2006). Accommodation with higher-order monochromatic aberrations corrected with adaptive optics. *Journal of the Optical Society of America A*.

[70] López-Gil N., Iglesias I., Artal P. (1998). Retinal image quality in the human eye as a function of the accommodation. *Vision Research*.

[71] López-Gil N., Rucker F. J., Stark L. R. (2007). Effect of third-order aberrations on dynamic accommodation. *Vision Research*.

[72] Gambra E., Sawides L., Dorronsoro C., Marcos S. (2009). Accommodative lag and fluctuations when optical aberrations are manipulated. *Journal of Vision*.

[73] Pérez-Merino P., Velasco-Ocana M., Martinez-Enriquez E., Marcos S. (2015). OCT-based crystalline lens topography in accommodating eyes. *Biomedical Optics Express*.

[74] Artal P., Berrio E., Guirao A., Piers P. (2002). Contribution of the cornea and internal surfaces to the change of ocular aberrations with age. *Journal of the Optical Society of America A*.

[75] Tabernero J., Benito A., Alcón E., Artal P. (2007). Mechanism of compensation of aberrations in the human eye. *Journal of the Optical Society of America A*.

[76] Applegate R. A., Ballentine C., Gross H., Sarver E. J., Sarver C. A. (2003). Visual acuity as a function of Zernike mode and level of root mean square error. *Optometry and Vision Science*.

[77] de Gracia P., Dorronsoro C., Gambra E., Marin G., Hernández M., Marcos S. (2010). Combining coma with astigmatism can improve retinal image over astigmatism alone. *Vision Research*.

[78] Atchison D. A., Pritchard N., Schmid K. L. (2006). Peripheral refraction along the horizontal and vertical visual fields in myopia. *Vision Research*.

[79] Lundström L., Gustafsson J., Unsbo P. (2009). Population distribution of wavefront aberrations in the peripheral human eye. *Journal of the Optical Society of America A*.

[80] Osuagwu U. L., Suheimat M., Atchison D. A. (2017). Peripheral aberrations in adult hyperopes, emmetropes and myopes. *Ophthalmic and Physiological Optics*.

[81] Dorronsoro C., Barbero S., Llorente L., Marcos S. (2003). On-eye measurement of optical performance of rigid gas permeable contact lenses based on ocular and corneal aberrometry. *Optometry and Vision Science*.

[82] Lopes-Ferreira D., Fernandes P., Queirós A., González-Meijome J. M. (2018). Combined effect of ocular and multifocal contact lens induced aberrations on visual performance: center-distance versus center-near design. *Eye & Contact Lens: Science & Clinical Practice*.

[83] Martin J. A., Roorda A. (2003). Predicting and assessing visual performance with multizone bifocal contact lenses. *Optometry and Vision Science*.

[84] Fedtke C., Sha J., Thomas V., Ehrmann K., Bakaraju R. C. (2017). Impact of spherical aberration terms on multifocal contact lens performance. *Optometry and Vision Science*.

[85] Kollbaum P. S., Dietmeier B. M., Jansen M. E., Rickert M. E. (2012). Quantification of ghosting produced with presbyopic contact lens correction. *Eye & Contact Lens: Science & Clinical Practice*.

[86] Fedtke C., Ehrmann K., Thomas V., Bakaraju R. C. (2016). Association between multifocal soft contact lens decentration and visual performance. *Clinical Optometry*.

[87] Pérez-Merino P., Marcos S. (2018). Effect of intraocular lens decentration on image quality tested in a custom model eye. *Journal of Cataract & Refractive Surgery*.

[88] El-Nimri N. W., Walline J. J. (2017). Centration and decentration of contact lenses during peripheral gaze. *Optometry and Vision Science*.

[89] Davis R. L. (2016). Determining multifocal parameters for a better fit. *Review of Optometry*.

[90] Plainis S., Ntzilepis G., Atchison D. A., Charman W. N. (2013). Through-focus performance with multifocal contact lenses: effect of binocularity, pupil diameter and inherent ocular aberrations. *Ophthalmic and Physiological Optics*.

[91] Koh S. (2018). Irregular astigmatism and higher-order aberrations in eyes with dry eye disease. *Investigative Opthalmology & Visual Science*.

[92] Montesmico R., Alió J. L., Muñoz G. (2004). Postblink changes in total and corneal ocular aberrations. *Ophthalmology*.

[93] Montés-Micó R., Alió J. L., Charman W. N. (2005). Dynamic changes in the tear film in dry eyes. *Investigative Ophthalmology & Visual Science*.

[94] Li K. Y., Yoon G. (2006). Changes in aberrations and retinal image quality due to tear film dynamics. *Optics Express*.

[95] Koh S., Maeda N., Kuroda T. (2002). Effect of tear film break-up on higher-order aberrations measured with wavefront sensor. *American Journal of Ophthalmology*.

[96] Wolffsohn J. S., Davies L. N. (2019). Presbyopia: effectiveness of correction strategies. *Progress in Retinal and Eye Research*.

[97] Fernandes P. R. B., Neves H. I. F., Lopes-Ferreira D. P., Jorge J. M. M., González-Meijome J. M. (2013). Adaptation to multifocal and monovision contact lens correction. *Optometry and Vision Science*.

[98] Richdale K., Mitchell G. L., Zadnik K. (2006). Comparison of multifocal and monovision soft contact lens corrections in patients with low-astigmatic presbyopia. *Optometry and Vision Science*.

[99] Gupta N., Naroo S. A., Wolffsohn J. S. (2009). Visual comparison of multifocal contact lens to monovision. *Optometry and Vision Science*.

[100] García-Lázaro S., Ferrer-Blasco T., Radhakrishnan H., Cerviño A., Charman N. W., Montés-Micó R. (2012). Visual function through 4 contact lens-based pinhole systems for presbyopia. *Journal of Cataract & Refractive Surgery*.

[101] Llorente-Guillemot A., García-Lazaro S., Ferrer-Blasco T., Perez-Cambrodi R. J., Cerviño A. (2012). Visual performance with simultaneous vision multifocal contact lenses. *Clinical and Experimental Optometry*.

[102] Madrid-Costa D., García-Lázaro S., Albarrán-Diego C., Ferrer-Blasco T., Montés-Micó R. (2013). Visual performance of two simultaneous vision multifocal contact lenses. *Ophthalmic and Physiological Optics*.

[103] Bakaraju R. C., Tilia D., Sha J. (2018). Extended depth of focus contact lenses vs. two commercial multifocals: part 2. Visual performance after 1 week of lens wear. *Journal of Optometry*.

[104] Sha J., Bakaraju R. C., Tilia D. (2016). Short-term visual performance of soft multifocal contact lenses for presbyopia. *Arquivos Brasileiros de Oftalmologia*.

[105] Tilia D., Munro A., Chung J. (2017). Short-term comparison between extended depth-of-focus prototype contact lenses and a commercially-available center-near multifocal. *Journal of Optometry*.

[106] Ferrer-Balsco T., Madrid-Costa D. (2010). Stereoacuity with simultaneous vision multifocal contact lenses. *Optometry and Vision Science*.

[107] Diec J., Tilia D., Naduvilath T., Bakaraju R. C. (2017). Predicting short-term performance of multifocal contact lenses. *Eye & Contact Lens: Science & Clinical Practice*.

[108] Pesudovs K., Hazel C. A., Doran R. M., Elliot D. B. (2004). The usefulness of Vistech and FACT contrast sensitivity charts for cataract and refractive surgery outcomes research. *British Journal of Ophthalmology*.

[109] Ferrer-Blasco T., Madrid-Costa D. (2011). Stereoacuity with balanced presbyopic contact lenses. *Clinical and Experimental Optometry*.

[110] Buckhurst P. J., Wolffsohn J. S., Gupta N., Naroo S. A., Davies L. N., Shah S. (2012). Development of a questionnaire to assess the relative subjective benefits of presbyopia correction. *Journal of Cataract & Refractive Surgery*.

[111] Wang B., Ciuffreda K. J. (2006). Depth-of-focus of the human eye: theory and clinical implications. *Survey of Ophthalmology*.

[112] Kingston A. C., Cox I. G. (2013). Predicting through-focus visual acuity with the eye’s natural aberrations. *Optometry and Vision Science*.

[113] Madrid-Costa D., Tomás E., Ferrer-Blasco T., García-Lázaro S., Montés-Micó R. (2012). Visual performance of a multifocal toric soft contact lens. *Optometry and Vision Science*.

[114] García-Lázaro S., Ferrer-Blasco T., Radhakrishnan H., Albarrán-Diego C., Montés-Micó R. (2012). Visual comparison of an artificial pupil contact lens to monovision. *Optometry and Vision Science*.

[115] Bradley A., Rahman H. A., Soni P. S., Zhang X. (1993). Effects of target distance and pupil size on letter contrast sensitivity with simultaneous vision bifocal contact lenses. *Optometry and Vision Science*.

[116] García-Lázaro S., Ferrer-Blasco T., Radhakrishnan H., Albarrán-Diego C., Montés-Micó R. (2014). Artificial pupil versus contralateral balanced contact lens fit for presbyopia correction. *Arquivos Brasileiros de Oftalmologia*.

[117] Faria-Ribeiro M., Amorim-de-Sousa A., González-Méijome J. M. (2018). Predicted accommodative response from image quality in young eyes fitted with different dual-focus designs. *Ophthalmic and Physiological Optics*.

[118] Aller T. A., Liu M., Wildsoet C. F. (2016). Myopia control with bifocal contact lenses: a randomized clinical trial. *Optometry and Vision Science*.

[119] Sankaridurg P., Bakaraju R. C., Naduvilath T. (2019). Myopia control with novel central and peripheral plus contact lenses and extended depth of focus contact lenses: 2 year results from a randomised clinical trial. *Ophthalmic and Physiological Optics*.

[120] Allen P. M., Radhakrishnan H., Price H. (2013). A randomised clinical trial to assess the effect of a dual treatment on myopia progression: the cambridge anti-myopia study. *Ophthalmic and Physiological Optics*.

[121] Pomeda A. R., Pérez-Sánchez B., Cañadas Suárez M. d. P., Prieto Garrido F. L., Gutiérrez-Ortega R., Villa-Collar C. (2018). MiSight assessment study Spain: adverse events, tear film osmolarity, and discontinuations. *Eye & Contact Lens: Science & Clinical Practice*.

[122] Ruiz-Pomeda A., Pérez-Sánchez B., Valls I., Prieto-Garrido F. L., Gutiérrez-Ortega R., Villa-Collar C. (2018). MiSight assessment study Spain (MASS). A 2-year randomized clinical trial. *Graefe’s Archive for Clinical and Experimental Ophthalmology*.

[123] Chamberlain P., Peixoto-de-Matos S. C., Logan N. S., Ngo C., Jones D., Young G. (2019). A 3-year randomized clinical trial of MiSight lenses for myopia control. *Optometry and Vision Science*.

[124] Bakaraju R. C., Ehrmann K., Ho A. (2018). Extended depth of focus contact lenses vs. two commercial multifocals: part 1. Optical performance evaluation via computed through-focus retinal image quality metrics. *Journal of Optometry*.

[125] Lam C. S. Y., Tang W. C., Tse D. Y.-Y., Tang Y. Y., To C. H. (2014). Defocus incorporated soft contact (DISC) lens slows myopia progression in Hong Kong Chinese schoolchildren: a 2-year randomised clinical trial. *British Journal of Ophthalmology*.

[126] Cheng X., Xu J., Chehab K., Exford J., Brennan N. (2016). Soft contact lenses with positive spherical aberration for myopia control. *Optometry and Vision Science*.

[127] Ruiz-Pomeda A., Fernandes P., Amorim-de-Sousa A. (2019). Light disturbance analysis in the controlled randomized clinical trial MiSight assessment study Spain (MASS). *Contact Lens and Anterior Eye*.

[128] Santolaria-Sanz E., Cerviño A., González-Méijome J. M. (2016). Corneal aberrations, contrast sensitivity, and light distortion in orthokeratology patients: 1-year results. *Journal of Ophthalmology*.

[129] Martins C., Amorim-De-Sousa A., Faria-Ribeiro M., Pauné J., González-Méijome J. M., Queirós A. (2019). Visual performance and high-order aberrations with different contact lens prototypes with potential for myopia control. *Current Eye Research*.

[130] Faria-Ribeiro M., González-Méijome J. M. (2019). Multifocal contact lenses: towards customisation?. *Ophthalmic and Physiological Optics*.

[131] Walline J. J., Lindsley K. B., Vedula S. S. (2011). Interventions to slow progression of myopia in children. *Cochrane Database Systematic Reviews*.

